# A cross‐sectional study of online learning during the COVID‐19 pandemic: Student perceptions

**DOI:** 10.1002/hsr2.1946

**Published:** 2024-03-13

**Authors:** Gjergji Koja, Erjona Abazaj

**Affiliations:** ^1^ Department of Clinical Subjects University “Alexander Xhuvani” Elbasan Albania; ^2^ Department of Epidemiology and Control of Infectious Diseases Institute of Public Health Tirana Albania

**Keywords:** difficulties, health problems, negative perceptions, online learning, students

## Abstract

**Background:**

Severe acute respiratory syndrome coronavirus 2 caused an unprecedented pandemic for more than 2 years, affecting the lifestyle health, and education systems at the global level. During this long pandemic period, online learning was considered the only secure learning alternative. Multiple studies have demonstrated positive or negative perceptions related to online teaching.

**Aim:**

Through this study, we identified the perceptions and different challenges that online learning brought to the education of the students of Alexander Xhuvani University, Elbasan, focusing on their achievements.

**Method:**

This cross‐sectional study was conducted by Alexander Xhuvani University from March to May 2021. A questionnaire that assessed students' perceptions related to the effectiveness of distance learning was used for this study. This questionnaire had a Cronbach *α* value of 0.760. Students from two faculties were welcomed to be part of this survey and to fill out the questionnaire on the Google platform. The data of the online questionnaire was made in Excel format and then analyzed using the statistical program SPSS version 20.0.

**Results:**

Overall, 350 students were included in the study about 268 (76.6%) belong to the female gender, and most students 239 (68.3%) belong to the age group of 18–20 years. It is worth mentioning that 271 (77.4%) had a negative perception related to e‐learning. Approximately, 201 (57.4%) of students encountered difficulties with the internet, 49 (14%) of cases reported a lack of technological equipment, and 111 (31.7%) encountered distraction while using the phone to listen to the online lesson. Problems with visual images and audio were also reported. As advantages, we mention freer time and greater flexibility in the learning process. Changes in health and management of online learning were observed in 123 (35.1%) of students, where loss of time and lack of concentration to learn, sleep problems, stress, anxiety, and fear were encountered the most.

**Conclusions:**

This is the first study in university education to assess students' perceptions and results achieved by online learning during our country's pandemic. Our study's findings show that 271 (77.4%) of participants have a negative perception of online learning. The most common challenges among students were Internet speed, a lack of technological equipment, and Internet disconnection. In terms of expectation fulfillment during online learning and assessment results, 245 (70%) of students' expectations were unsatisfied and unmet. These findings will address the gaps in the literature and may influence future online learning design in our country.

## 1 INTRODUCTION

Education at all levels serves as a cornerstone for the establishment and emancipation of a state. The more educated a country is, the more developed and powerful that country will be. Designing appropriate curricula and programs for each educational level, as well as creating the necessary and adequate conditions for teaching, are significant challenges for stakeholders and institution heads. Since the commencement of the educational system, education has always been founded on an interactive relationship between the teacher and the student, which takes place in traditional classrooms face‐to‐face. Even though Barrot et al. state that educational systems around the world have undergone substantial changes as a result of the ever‐expanding influence of technology,^1^ the advent of online learning as a new alternative where teachers virtual lessons in an interactive manner, exhibits materials effectively, and enable interaction and collaboration among students, has been more visible in the last two decades.^2^, ^3^

Almost all educational systems in Albania are focused on traditional learning methods, but during the last few years, it seems that some colleges, largely private, have begun to implement mixed learning.^4^ On the other hand, many others, particularly public education, are thoroughly embedded in the traditional manner of teaching. For more than 2 years, the abrupt COVID‐19 epidemic triggered by the Severe acute respiratory syndrome coronavirus 2 virus has caused an unprecedented threat to global public health by making people unable to live a better lifestyle and education.^5^ Many countries around the world took extreme measures accompanied by social distancing and restrictions on contact among people.^6^ One of the fields directly affected by this pandemic was education.^7^ During the quarantine time, almost all educational institutions, including universities, suspended teaching and began to prepare quickly to switch to the online learning‐teaching process. Distance learning appeared as the only secure option worldwide.^8^ As a result, traditional classroom learning almost immediately paved the way for more modern virtual classroom learning.^9^ According to Amer and Ouhida, during virtual teaching, the teacher and the students did not meet face to face each other but met via the Internet by using different platforms of learning, like WhatsApp, Zoom Meeting, Google Meet, Ed Link, and many more.^10^ Even though this transition was viewed as a revolution in the education system by certain countries, on the other part, this learning system emphasized the necessity of alternative teaching and learning models and methodologies, particularly in online teaching and learning resources.^11^, ^12^, ^13^, ^14^, ^15^

The situation caused by the COVID‐19 pandemic and the online learning transition found both the educational staff and the students unprepared. Many of them were sceptical, while some others had negative attitudes toward online learning, whereas other students had the perception that online learning would mean low quality of education. Multiple studies come to the same conclusion regarding positive or negative perceptions of students on online learning.^9^, ^16^, ^17^, ^18^, ^19^, ^20^ In several studies, it was found that during online learning, students did not manage to have maximum concentration,^1^ the distraction was easier at home,^20^ they had deficiencies or technical problems with the Internet, computer equipment,^21^ and most importantly, they lacked direct relationships with friends and fellow faculty.^22^, ^23^ Thus, in a study conducted with 1207 students in Pakistan with the inclusion of 31 questions, the majority of students included in this study were dissatisfied with online learning both in terms of course management and the quality of teaching or information provided during classes. online.^24^ Pei and Wu found out in their systematic study^25^ that online learning is at least as effective as face‐to‐face learning for undergraduate medical students.

In addition to negative perceptions about online learning, many other studies have highlighted a rather positive impact compared to face‐to‐face classroom learning. In two different studies, Cheng et al.^26^ as well as Yun et al., ^21^ ^27^ reported that students had positive thoughts. Based on the findings of previous research, students understood that online learning had advantages, such as increased flexibility in learning; saving time and money for traveling to universities; more space to express their learning speed, and health security to COVID‐19 due to lack of contact with others.^26^, ^27^ Tuma et al., in a cross‐section study involving students and instructors, found that more than 30% of the students and more than half of the instructors rated online learning as equal to or superior to face‐to‐face learning in traditional classes.^14^ Based on these distance learning experiences worldwide expressed in many studies and at the same time lack of experience of Albanian students during online learning, this study took a unique opportunity to investigate any such perception in students at Elbasan University. Through this study, we identified the perceptions and different challenges that online learning brought to the education of the students of Alexander Xhuvani University, Elbasan, focusing on their achievements.

## 2 METHODS

### 2.1 Study design

This cross‐sectional study was undertaken to investigate the perception and various challenges that online learning introduces to the education of students, focusing on their achievements by using a self‐administered online questionnaire. The research was conducted by the Alexander Xhuvani University from March to May 2021. As a result, the university board approved this study. Reviewing the literature, determining the purpose of the study and raising research questions, determining the research instrument, analyzing the data and preparing the results, and discussing and comparing the findings were the exact tasks that arose from the beginning of the conception of this study. The questionnaire was validated in stages.

### 2.2 The study context of the study population

The research was carried out at Aleksander Xhuvani University in Elbasan, with the participation of two faculties (the Faculty of Technical Medical Sciences and the Faculty of Education Sciences). This university offers education in two primary programs: bachelor's and master's (professional and scientific master's). Students in these faculties study in a variety of fields. For example, in the faculty of medical and technical sciences, students study Nursing, Midwifery, Speech Therapy, Imaging Technician, and Laboratory Technician. While the Faculty of Education Sciences has branches such as pre‐school and higher cycle teachers, psychology, social work, and so on. The total number of students in both faculties and study programs exceeds 3500.

### 2.3 Data collection tools

The study was guided by the following five research questions^1^: What is students' perception of online learning based on the effectiveness of teaching during the COVID‐19 pandemic?^2^ What challenges did virtual technologies pose for online learning during the COVID‐19 pandemic?^3^ What were the advantages and disadvantages of online learning and teaching during the COVID‐19 pandemic?^4^ What were the expectations in terms of achievements and unfulfillment of results?^5^ Did the transition to online study during the COVID‐19 pandemic affect their health?

Initially, we tested the validity of our questions by sending them to some experts in the field for their feedback. Second, the internal consistency of the questionnaire was assessed, and the result was 0.76. Third, the questionnaire was delivered to a pilot sample of 20 students, 10 from each faculty, and these students were asked about the clarity of the questionnaire. All student perceptions were used while designing the final questionnaire.

### 2.4 Defining the research instrument: Questionnaire development

During COVID‐19, a questionnaire was drafted based on various prior studies that assessed students' perceptions related to the effectiveness of distance learning.^28^, ^29^, ^30^, ^31^, ^32^, ^33^ The questionnaire consisted of three evaluation units. In the first part, the questionnaire began with the demographic questions of the participating students, which included questions about gender, age, place of residence (village/city), faculty (technical; medical science or education sciences), study degree (bachelor/master) and academic year (from first year until 5 years). The second part was focused on the perception of online learning and teaching and the various challenges that students encountered during the online learning transition. Some of the questions in the second section were dichotomous (yes/no, female/male), others were ordinal, and some other questions, such as the challenges encountered, were initially left open to allow students to express their perceptions and challenges encountered during distance learning. In the third section, doubts were raised about online learning engagement and whether it was equivalent to traditional learning. More precisely we asked about attendance during the e‐learning; the differences encountered in teaching from different professors; the administration of online exams, what were their results; expectations related to exams; the benefits or drawbacks of online learning, and, finally, the problems encountered with mental health. For this part of the questionnaire, the students had the opportunity to circle one or more answers.

Following careful reflection, the questions were reworked and restructured to best suit our needs and elicit the greatest potential responses. For some of the questions, such as questions relating to the appraisal of problems and disadvantages or advantages (after practically all of the relevant points were gathered) we opted to utilize the Likert‐type scale. Taking into consideration the adoption of the final form, the questionnaire was retested on 20 more students, and the Cronbach alpha was calculated using statistical software version 26.0. This questionnaire had a Cronbach *α* value of 0.760 (95% confidence interval [CI]; 0.71−0.87).

### 2.5 Sampling collection

Following the completion of the final form of the questionnaires, the forms were entered into the Google platform, and all students from the two aforementioned faculties were welcomed to participate in the survey during their spare hours, via the teaching staff. The size of our sample was computed statistically using the Yamane formula,^34^ with a confidence level of 95% and an acceptable margin of error of 5%. The formula is:

$$n=N/(1+{\mathrm{Ne}}^{2}),$$

where *n* = the sample size; *N* = the population size. Finally, this study used the questionnaires completed by 350 students. The survey's introduction emphasized that participation was entirely voluntary and that their anonymous replies would be utilized in aggregate form only for this research purpose. Students from several fields were recruited for this survey.

To maintain anonymity and data confidentiality, it did not require participants to log in before taking the survey. The procedure did not collect IP addresses and students who did not desire to participate in this study could not fulfill the questionnaires.

**Figure 1:**
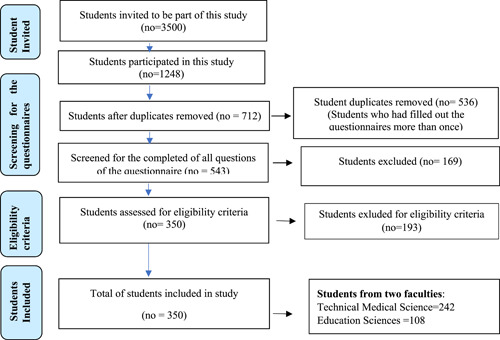
Flowchart for students' participation in the study.

The active engagement of students, regardless of gender, aged 18–26 years, regardless of study cycle or year of study, was used as an inclusive criterion in this paper (Figure 1). The questionnaire link was sent to students via email, WhatsApp, Messenger, and other messaging and social media platforms. During the availability period, the data was stored on the Google platform, and the final data set was exported as a Microsoft Excel file.

### 2.6 Statistical analysis

SPSS statistical software version 26 (SPSS Inc) was used to evaluate the data obtained in this study. The descriptive analysis provided the demographic profile of students who responded to the questionnaires. The independent variables were socio‐demographic factors, and the dependent variable was the perception and expectation of their achievements. The categorical variables were presented as frequencies and percentages. The perceptions, and expectations of their achievements were dichotomized into two responses, “positive,” or “negative” perceptions and satisfactory and unsatisfactory expectation achievements. Chi‐square was used to compare the variables, while with logistic regression, we investigated the potential predictors of expectations of their achievements. *p*‐value <  0.05 was considered statistically significant.

### 2.7 Ethical considerations

The Ethics Committee of Aleksander Xhuvani University granted ethical approval for this cross‐sectional investigation. Furthermore, from all colleagues and the head of faculty institution permission, was obtained. Also, during this study, the fulfillment of the standards of the Declaration of Helsinki was ensured. This is an anonymous online questionnaire. Student participation in the study was voluntary, they could withdraw from this study at any time and no data was collected except gender, faculty, and year of study.

## 3 RESULTS

### 3.1 Demographic data

The first table shows the demographic data of the students participating in this study. As can be seen, the most predominant part, or a little more than ¾ of the students belong to the female gender 268 (76.6%), and approximately ¼ of them were male. Regarding the ages of the participants, 239 (68.3%) of the students were in the age group of 18–20 years, 88 (25.1%) were in the age group of 21–24 years, and only 23 (6.6%) were aged ≥25 years. Most of the students, 196 (56%), lived in the city (including the cities of Elbasan, Pogradec, Korça Tirana, etc), and 44% lived in the countryside.

According to the findings, 242 (69.1%) of them referred that they were part of the Faculty of Technical Medical Sciences and 108 (30.8%) referred that they were part of the Faculty of Education Sciences. Regarding the study cycle, 243 (69.4%) of students obtained a bachelor's degree, while 107 (30.6%) obtained a master's degree (professional and scientific master together). In the first year of studies, 112 (32%), in the second year, 80 (22.9%), in the third year, 51 (14.6%), in the fourth year, 70 (20%), and in the fifth year, 37 (10.6%) of students (Table 1).

**Table 1:** Demographic data of students participating in the study.

Variables	Alternatives	Frequency	Percent
Gender	Female	268	76.6
	Male	82	23.4
Age	18–20 years old	239	68.3
	21–24 years old	88	25.1
	≥25 years old	23	6.6
Residence	Village	154	44
	City	196	56
Faculties	Technical medical sciences	242	69.1
	Education sciences	108	30.8
Study degree	Bachelor	243	69.4
	Master	107	30.6
Academic year	I	112	32
	II	80	22.9
	II	51	14.6
	IV	70	20
	V	37	10.6

### 3.2 Data analysis related to perception, challenges, and achievements

The second table presents detailed information about the progress of online learning according to the perceptions of students. It is worth emphasizing from the beginning that online learning among the students participating in this study was not suitable. Only 79 (22.6%) of them gave a positive perception of online learning the others had a negative impact 271 (77.4%).

The majority of students about 298 (85.1%) reported that during online learning they have faced difficulties. Approximately 201 (57.4%) of cases referred to problems related to the speed and disconnections of the Internet, in 49 (14%) of cases the lack of technological equipment, 111 (31.7%) noticed distraction while using the phone to listen to the online lesson, 142 (40.6%) noticed a problem with the audio, 103 (29.4%) noticed problems with the visual appearance (*p* < 0.05) (Table 1).

Regarding the frequency of Internet disconnection, 80 (20.6%) reported that during a lesson the Internet was disconnected one time, 154 (44%) reported that the Internet was disconnected two times (*p* < 0.05), and 73 (20.6%) reported that the Internet was disconnected at least three times. Meanwhile, the average time during which the lesson developed normally without being interrupted was 61 (17.4%) in 10 min, 176 (50.3%) in 20 min (*p* < 0.05), and 70 (20%) in 30 min or more.

Except challenges mentioned above, students have referred to the other problems. Thus, 64 (18.3%) stated that during the presentation there was ambiguity with the PowerPoint presentation, 120 (34.3%) said the time was insufficient and this was influenced by frequent internet disconnections, 138 (39.4%) had problems with reconnection, and 80 (22.9%) had other problems such as p.eg, problems with communication between professors and other students, not understanding the writing on the boards, the volume of the voice) (*p* < 0.05) (Table 2).

**Table 2:** Information related to the perception and development of online learning.

Questions	Alternatives	Frequency	Percentage
Has your learning and education been positively impacted by online learning compared to traditional teaching?	Yes^a^	79	22.6
	No	271	77.4
Have you encountered difficulties while learning online?	Yes^a^	298	85.1
	No	52	14.6
What difficulties have you encountered most often during online learning? (multiple choice question)	Internet speed problems and disconnections^a^	201	57.4
	Lack of technological equipment (computer) in the home environment	49	14
	During online learning, they often encountered difficulties with concentration	111	31.7
	Audio problems	142	40.6
	Visual appearance is not good	103	29.4
	None above	43	12.3
How frequent were these difficulties during a lesson?	One time	80	22.6
	Two times^a^	154	44
	Three times	73	20.6
What was the average time (within 1 h of teaching) when you had a lesson without any interruptions?	10 min	61	17.4
	20 min^a^	176	50.3
	30 min or more	70	20
What other difficulties have you faced while learning online?	The presentation slide in PowerPoint format is not very clear	64	18.3
	Not enough time	120	34.3
	Difficulty re‐connecting with learning^a^	138	39.4
	Others	80	22.9

^a^ Chi‐square *p*‐value significant (<0.05).

The next questionnaire is about describing and evaluating (with reasons for some of the questions) related to the online learning in which you participated. Based on student referrals, 210 (60%) have attended regularly just as during traditional instruction.

Regarding the evaluation of the commitment of their friends, 85 (24.3%) referred that it was the maximum commitment, 207 (59.1%) estimated that it was satisfactory and 58 (16.6%) it was low. Most of the students have positively evaluated online teaching by the professors. About 197 (56.3%) said that from the professors' side, there was no difference between online and traditional teaching (<0.05) (Table 3).

**Table 3:** Description and assessment of online learning by students.

Questions	Alternatives	Frequency	Percentage
Has your commitment during online learning been the same as your engagement in traditional learning?	Yes^a^	210	60
	No	140	40
How do you rate the commitment of other students during online learning compared to traditional learning?	Maximum	85	24.3
	Satisfactory	207	59.1
	Low	58	16.6
Has there been any difference in teaching by the professors during online learning, compared to the traditional one?	Yes	153	43.7
	No^a^	197	56.3
What is your perception about the development of online exams? Were they easier or harder?	Very good perception	65	18.6
	Satisfactory perception	99	28.3
	I don't have a good perception^a^	143	40.9
	Neither	43	12.3
How were the results obtained from online learning?	Very high	60	17.1
	Satisfactory	181	51.7
	Not good at all	109	31.2
Did they meet your expectations?	Yes	105	30
	No^a^	245	70
What were the advantages and disadvantages of online learning according to you? Explain in words.			
Regarding online learning, did you notice any changes in your health or the way you managed your normal days before online learning?	Yes	123	35.1
	No^a^	227	64.9
Explain changes			
What do you think needs to be improved in online learning delivery?			

^a^ Chi‐square *p*‐value significant (<0.05).

Regarding the way of giving online exams, 65 (18.6%) had a very good perception, 99 (28.3%) had a satisfactory perception, 143 (40.9%) did not have a good perception and 43 (12.3%) did not express themselves. Furthermore, 60 (17.1) reported that the results obtained from online learning were very high, 181 (51.7%) were satisfactory and 109 (31.1%) were not good at all (*p* < 0.05).

Regarding the fulfillment of expectations during online learning and the assessment results, 105 (30%) reported that expectations of achievements were satisfied and in 245 (70%) of students, expectations were unsatisfied and not met. Related to the advantages and disadvantages of online learning, most students have listed freer time, greater flexibility of the learning process, better conditions in the home environment, and not taking absences.

The disadvantages students have listed are problems with internet connection, lack of verbal communication with other students, greater distraction, limited interpersonal relationships, and not offering practical skills mainly for students of Technical Medical Sciences as well as subjective evaluation (Table 3).

Online learning has also been associated with changes in health or time management in 123 (35.1%) of students. The most common changes were sleep problems 5 (4.0%), stress 7 (5.7%), anxiety 6 (4.9%), fear of online learning and exam evaluation 12 (9.8%), boredom and sadness 14 (11.4%), wasting time and not concentrating on learning 31 (25.2%) (<0.05).

Based on the in‐depth logistic regression analysis between some of the variables and the expectations of achievements on online learning and online assessments by students, a significant relationship is observed for gender, residence, perception toward online learning, assessment in exams, and changes in health. The value of *p* was significantly <0.05% (Table 4).

**Table 4:** Logistic regression of student's expectations.

Variables	Odds ratio	Reliability interval (95% confidence interval)	*p*‐value
Gender	1.4	(1.03–2.2)	0.03
Age	1.1	(0.6–1.9)	0.23
Residence	2.2	(1.2–3.4)	0.001
Faculty	0.8	(0.1–1.6)	0.90
Study degree	0.4	(0.08–1.5)	0.54
Academic year	0.9	(0.5–1.7)	0.91
Perceptions toward online learning and education compared to traditional teaching	2.5	(1.2–3.6)	0.006
Assessment in exams	0.6	(0.01–0.9)	0.01
Changes in health	1.6	(1.1–2.6)	0.04
Time management as a result of online learning	1.2	(0.8–1.8)	0.11

## 4 DISCUSSION

Nelson Mandela stated, “Education is the most powerful weapon you can use to change the world.” The development of a country is mainly based on its people and resources, where education is responsible for shaping a person.^35^ Therefore, education is the backbone of any country, it plays a crucial role in technological development and imparts various skills, values, and awareness.

During the COVID‐19 pandemic, our lives changed in an instant. All of us already had to adapt to the new quarantine and social distancing measures. In the beginning when the virus spread, information about the routes of transmission, the speed of transmission, the lack of adequate treatment, and the lack of an efficient vaccine were very few.^36^ This led to the finding of many suitable alternatives to continue the course of life.^37^ One of the adaptations was the transition of the entire educational system from traditional learning to distance learning. For many of the educational staff, this transition was a tremendous effort, as the time available to prepare and adapt to online teaching was relatively short.^38^ Furthermore, the students also faced great difficulties, since the end of their student life, returning to their families, as well as the lack of infrastructure necessary for this type of learning.^38^, ^39^

Many studies have evidenced different aspects of online learning before and after the COVID‐19 pandemic time.^40^, ^41^, ^42^, ^43^, ^44^ On the other hand, there is a lack of research on empirically investigated identifying and understanding factors, that impacted directly students during online classes, and how they have affected their achievements. Over three‐quarters of the participants in this study were female, and over half of them were in the 18–20 age range. It is important to note that the majority of participants attended the Faculty of Technical Medical Sciences for their education.

Distance learning is a useful tool for overcoming unforeseen emergencies, such as the COVID‐19 pandemic, according to Ayebi‐Arthur.^44^ In reference to the perception of online learning, Kulal and Nayak emphasized that “online learning is considered a fun way to learn and it has a positive impact on both students and teachers.”^45^ Also, another study was conducted by Meccawy et al. at King Abdulaziz University during the pandemic, to gather information on faculty staff and student perceptions of online learning. This study found that students had more positive perceptions of online learning than faculty teaching staff.^46^ However, a survey carried out in Libya revealed that 64.7% of the 3348 student participants in the study disagreed with online learning.^47^ Moreover, another study conducted by Almahasees et al. at the Arab Region Universities, with the inclusion of 50 faculty members and 280 students, reported that the effectiveness of online learning was less effective than face‐to‐face learning and teaching.^9^ Even in our study, a negative impact regarding online learning on the part of 77.4% of students was observed.

The most predominant part of students (85.1%) included in our study, encountered difficulties while learning online. Based on this finding we have seen that other research presented a lot of difficulties and challenges during online learning. So, several disadvantages related to online learning have been brought to light by the same study by Almahasees et al. Teachers and students reported that they had challenges with adaptability, poor listening skills, a lack of motivation and involvement, technical and Internet‐related problems, and concerns about data security and privacy when learning online.^9^ Subedi et al. found that almost half of the participants (48.1%) were worried about online learning because of internet problems. More than half of the students (63.2%) were affected by the lack of electricity, and only (64.4%) of the students had access to the Internet for online learning.^48^ Similar to this, in the Tuma et al. study,^14^ students first expressed satisfaction with online assessments; however, (69%) of students reported greater challenges with online learning, which were primarily brought on by the accessibility of technology, the stability of their Internet connections, and their weariness from learning online. The findings of our study are in the same line with previous studies. Based on analyzed data, in this study many of the students (57.4%) encountered problems with the Internet (such as Internet speed problems and disconnections), (14%) reported a lack of computer or access to technological devices in their families, about (31.7%) encountered difficulties with concentration during online learning, (40.6%) encountered audio problems almost all the time of lectures, and (29.4%) encountered visual appearance problems, where the view is not good during the lessons. Except for the difficulties presented above, students encountered some other challenges. So, about (18.3%) reported problems with presentation slides in PowerPoint format, because sometimes the view of slides is not very clear. Moreover, (34.3%) did not have enough time to interact with the lecturers and students, and they failed to ask questions when had unclear things about the lesson. The biggest challenges encountered by the students were interruption of the internet and difficulty re‐connecting with learning.

The effectiveness and preferences of lecturers for both traditional and online learning methods have been assessed in many studies and research projects by researchers from a wide range of disciplines since the 2000s.^49^, ^50^, ^51^, ^52^, ^53^, ^54^

Even though these studies did not find any substantial changes in online teaching, they did find some minor variations depending on the professors' academic level or age.^49^, ^50^, ^51^, ^52^, ^53^, ^54^ In the current study, more than half of the students (56.3%) said they had not encountered any changes during the e‐teaching they even evaluated it at the same educational level as the traditional one. In many other studies, there are advantages and disadvantages of using both systems, according to the students' perception.^54^, ^55^, ^56^, ^57^, ^58^, ^59^, ^60^

The quality of distance learning and teaching is impacted by learning settings, according to a study by Selvaraj et al. that involved teachers and students in India.^60^ According to Bijeesh, more significant advantages of online learning were the lowest financial cost for both students and schools, saving of time, flexibility, comfort during learning, and better conditions in the house (if there is the necessary infrastructure for learning online), as well as most importantly immediate updates of the information provided online.^58^ According to the same author, the most important online learning disadvantage tended to highly distract students which meant poor performance on their part.

Furthermore, we should also mention the hidden costs related to the complicated technology that is required and the infrastructure (having a computer or device as well as the Internet connection) needed in students' homes.^58^ Gautam, in her article on eLearning, also mentioned the disadvantages of online learning. She has highlighted the disadvantages, “the sense of isolation, screen time management, teacher training regarding the newly built infrastructure, and poor or intermittent internet connection which can lead to poor online learning quality”.^59^ This can be detrimental to the teaching and learning process. The findings of our study were in the same line as the previous study. As regards advantages, many of the students emphasized the free time they had available, greater flexibility of the learning process, better conditions in the home environment, and not taking absences. As disadvantages, they highlighted problems with internet connection, the lack of interaction and no verbal communication with other students, greater distraction, limited interpersonal relationships, and no provision of practical skills.

Related to the achievements and failures during online learning, only 17.1% of students included in this study reported that arrived high‐level results, 51.7% arrived at a moderate level and 31.2% had low results in exams. Furthermore, in 70% of students, the distance learning and achievements did not live up to their expectations, and this was reflected in their low test scores. Additionally, some students expressed their doubts regarding the development of online exams, regardless of how easy or challenging they were. Following the exams, 18.6% had a good perception, that the tests were easier and comprehendible. Exam quality was rated as satisfactory by about 28.3% of respondents, while exams were rated as completely terrible by 40.9%. The main actors of educational systems such as students, parents, and teachers should give their contribution and devotion to all levels of education. Students and teachers may experience a variety of physical and psychological issues that have a direct impact on the process of learning and teaching during a challenging pandemic or significant national or international crises. No matter how radical the change may be, it must be handled extremely carefully because there will be future consequences.^37^ Rutkowska et al. referred in their study that (58%) of students are characterized by an increased level of stress, while (56%) showed symptoms of depression.

According to these researchers, isolation from friends and acquaintances, the negative impact on the level of knowledge, the decrease in motivation to learn, and the deterioration of grades were the main factors in the deterioration of students' health.^61^ Moreover, Gewalt et al. reported in their study that the COVID‐19 pandemic and the transition to online learning turned into a threat to physical health, mental health, and social well‐being. and the academic performance of the 917 students included in this study.^62^

In the current study, online learning among our students has been accompanied by changes referring to health or time management in 35.1% of students. Sleep problems, stress, anxiety, fear of online learning and exam evaluation, boredom, sadness, as well as loss of time, and lack of focus on learning were the most common problems. Moreover, it was also observed that male students exhibited a lower risk of distress compared to female students.

When they were asked about their perception of what to improve in e‐learning, the majority of students said that an explicit clear, and detailed study online learning curriculum should be used, which can lead to a more motivating learning orientation, increased satisfaction, and greater commitment to learning. Finally, before implementing online learning standards everyone must have access to the appropriate tools and equal learning facilities. The study's findings are particularly important for our country because the transition of classes from traditional to online classrooms happened overnight with no previous preparation on the part of the teaching staff or the students. According to the findings, the researchers acknowledge the importance of online learning in education during the pandemic and suggest that “adequate preparation, good curricula, and clear ideas, having adequate teaching platforms and an uninterrupted internet line, and student engagement activities” are key factors in improving educational quality. The study's conclusions direct us to take action and find better teaching options with the sole goal of enhancing student learning. This will encourage students to view instruction in a more positive and appealing light, resulting in a more knowledgeable and intellectually emancipated society. More investment in technology and basic infrastructure is required to allow unrestricted access to online classes for students and teachers from all walks of life. We anticipate that this study will serve as a foundation for future studies on a much larger size on the topics covered in this study, allowing us to enter a new frontier in the education sector.

## 5 CONCLUSION

This is the first study in university education to assess students' perceptions and results achieved by online learning during our country's pandemic. Our study's findings show that 77.4% of participants have a negative perception of online learning. The most common challenges among students (*p* = 0.05) were Internet speed, a lack of technological equipment, Internet disconnection, problems with PowerPoint presentations, a lack of time, and so on. According to 56.3% of academics, there is no difference between online and traditional instruction. In terms of expectation fulfillment during online learning and assessment results, 70% of students' expectations were unsatisfied and unmet. Some of the benefits included more free time, greater flexibility in the learning process, better conditions at home, and avoiding absences. Even though we investigated some of the factors that influence students' perceptions of e‐learning, more in‐depth studies with a larger number of participants and a broader scope across the country are required to demonstrate in greater detail which of the factors had the most influence on the student's achievements.

Students emphasize that, due to the unfavorable conditions in our nation, internet learning cannot replace traditional classroom learning. Furthermore, these findings indicate that distance learning in our country still needs improvement. It should be gradually integrated into our educational system, based on the country's infrastructure and student characteristics. Furthermore, student suggestions and grievances should be taken into account during future planning for the transition to online learning to avoid as many difficulties as possible, improve learning conditions, and raise student awareness. These findings address gaps in the literature and may influence future online learning design in our country.

## 6 LIMITATIONS

The students included in our study were only from two faculties of Aleksande Xhuvani University, therefore, the sample was not representative of students' perceptions at the national level for all universities, which may limit generalizability to other populations.

## AUTHOR CONTRIBUTIONS

**Gjergji Koja**: Conceptualization; data curation; formal analysis; funding acquisition; investigation; methodology; writing—original draft; writing—review and editing. **Erjona Abazaj**: Data curation; formal analysis; methodology; writing—original draft; writing—review and editing.

## CONFLICT OF INTEREST STATEMENT

The authors declare no conflict of interest.

## TRANSPARENCY STATEMENT

The lead author Erjona Abazaj affirms that this manuscript is an honest, accurate, and transparent account of the study being reported; that no important aspects of the study have been omitted; and that any discrepancies from the study as planned (and, if relevant, registered) have been explained.

## Data Availability

The data that support the findings of this study are available from the corresponding author upon reasonable request.
